# Necrotizing soft tissue infections in critically ill neutropenic patients: a French multicentre retrospective cohort study

**DOI:** 10.1186/s13613-023-01125-w

**Published:** 2023-04-28

**Authors:** Romain Arrestier, Anis Chaba, Asma Mabrouki, Clément Saccheri, Emmanuel Canet, Marc Pineton de Chambrun, Annabelle Stoclin, Muriel Picard, Florent Wallet, François Perier, Matthieu Turpin, Laurent Argaud, Maxens Decavèle, Nahéma Issa, Cyril Cadoz, Kada Klouche, Johana Cohen, Djamel Mokart, Julien Grouille, Tomas Urbina, Camille Hua, Olivier Chosidow, Armand Mekontso-Dessap, Elie Azoulay, Nicolas de Prost

**Affiliations:** 1 Service de Médecine Intensive Réanimation, Hôpitaux Universitaires Henri Mondor, Assistance Publique-Hôpitaux de Paris, CEDEX, Créteil, 94010 Paris, France; 2 Groupe de Recherche Clinique CARMAS, Faculté de Santé de Créteil, Université Paris Est Créteil, CEDEX, Créteil, 94010 Paris, France; 3 INSERM, IMRB, Université Paris Est Créteil, CEDEX, Créteil, 94010 Paris, France; 4 Assistance Publique-Hôpitaux de Paris, Service de Médecine Intensive Réanimation, Hôpitaux Universitaires Paris-Centre, Hôpital Cochin, Paris, France; 5 Service de Médecine Intensive et Réanimation, Hôpital Saint-Louis, AP-HP, Paris, France; 6 Service de Médecine Intensive Réanimation, Hôpital Archet 1, Centre Hospitalier Universitaire de Nice, Nice, France; 7 Service de Médecine Intensive Réanimation, Centre Hospitalier Universitaire de Nantes, Nantes, France; 8 Service de Médecine Intensive-Réanimation, Assistance Publique-Hôpitaux de Paris (APHP), Hôpital La Pitié-Salpêtrière, Sorbonne Université, Paris, France; 9 Service de Médecine Intensive Et Réanimation, Institut Gustave Roussy, Villejuif, France; 10 Service de Réanimation Polyvalente, Centre Hospitalier Universitaire de Toulouse, Institut Universitaire du Cancer de Toulouse Oncopole, Toulouse, France; 11 Hospices Civils de Lyon, Service d’anesthésie, Médecine Intensive, Réanimation, CHU Lyon Sud, Pierre-Bénite, France; 12 Réanimation Médico-Chirurgicale, Hôpital André Mignot, Centre Hospitalier de Versailles, Le Chesnay-Rocquencourt, France; 13 Sorbonne Université, Assistance Publique-Hôpitaux de Paris, Service de Médecine Intensive Réanimation, Hôpital Tenon, Paris, France; 14 Service de Médecine Intensive, Réanimation, Hôpital Edouard Herriot, Hospices Civils de Lyon, Lyon, France; 15 AP-HP, Sorbonne Université, Site Pitié-Salpêtrière, Service de Pneumologie, Médecine Intensive et Réanimation, Département R3S, Hôpital Pitié-Salpêtrière, Paris, France; 16 Médecine Intensive Réanimation, Hôpital Saint-André, CHU Bordeaux, France; 17 Réanimation Polyvalente, CHR Metz-Thionville Hôpital de Mercy, Metz, France; 18 Service de Médecine Intensive et Réanimation, CHU Montpellier, France; 19 Service de Médecine Intensive et Réanimation, Groupe Hospitalier Intercommunal le Raincy Montfermeil, Montfermeil, France; 20 Unité Traitement Soins Intensifs, Institut J.Paoli, I.Calmettes, Marseille, France; 21 Service de Réanimation Polyvalente, Centre Hospitalier Simone Veil, Blois, France; 22 Service de Médecine Intensive et Réanimation, Hôpital Saint-Antoine, Assistance Publique-Hôpitaux de Paris, Paris, France; 23 Service de Dermatologie, Hôpitaux Universitaires Henri Mondor, Assistance Publique-Hôpitaux de Paris, CEDEX, Créteil, 94010 Paris, France; 24 Epidemiology in Dermatology and Evaluation of Therapeutics (EpiDermE), EA 7379, Université Paris Est Créteil (UPEC), Créteil, France

**Keywords:** Necrotizing soft tissue infection, Neutropenic, Sepsis, Immunocompromised, Intensive care

## Abstract

**Background:**

Necrotizing soft tissue infections (NSTIs) are rare life-threatening bacterial infections. Few data are available regarding neutropenic patients with NSTIs. Our objectives were to describe the characteristics and management of neutropenic patients with NSTIs in intensive care units (ICUs). We conducted a retrospective multicentre cohort study in 18 ICUs between 2011 and 2021. Patients admitted with NSTIs and concomitant neutropenia at diagnosis were included and compared to non-neutropenic patients with NSTIs. The relationship between therapeutic interventions and outcomes was assessed using Cox regression and propensity score matching.

**Results:**

76 neutropenic patients were included and compared to 165 non-neutropenic patients. Neutropenic patients were younger (54 ± 14 vs 60 ± 13 years, *p* = 0.002) and had less lower limb (44.7% vs 70.9%, *p* < 0.001) and more abdomino-perineal NSTIs (43.4% vs 18.8%, *p* < 0.001). Enterobacterales and non-fermenting gram-negative bacteria were the most frequently isolated microorganisms in neutropenic patients. In-hospital mortality was significantly higher in neutropenic than in non-neutropenic patients (57.9% vs 28.5%, *p* < 0.001). Granulocyte colony-stimulating factor (G-CSF) administration was associated with a lower risk of in-hospital mortality in univariable Cox (hazard ratio (HR) = 0.43 95% confidence interval (CI) [0.23–0.82], *p* = 0.010) and multivariable Cox (adjusted HR = 0.46 95% CI [0.22–0.94], *p* = 0.033) analyses and after overlap propensity score weighting (odds ratio = 0.25 95% CI [0.09; 0.68], *p* = 0.006).

**Conclusions:**

Critically ill neutropenic patients with NSTIs present different clinical and microbiological characteristics and are associated with a higher hospital mortality than non-neutropenic patients. G-CSF administration was associated with hospital survival.

**Supplementary Information:**

The online version contains supplementary material available at 10.1186/s13613-023-01125-w.

## Background

Necrotizing soft tissue infections (NSTIs) are life-threatening infections characterized by skin, subcutaneous tissue, fascia or muscle necrosis. NSTIs are rare diseases, with an incidence varying between 0.2 and 6.9 per 100,000 persons-years [1–3]. Organ failure is common with almost 50% of patients requiring intensive care unit (ICU) admission [4]. Mortality rates range from 10 to 30% [5] and increase with initial misdiagnosis. Early management relies on multidisciplinary care including early surgical debridement and broad-spectrum antibiotherapy.

Immunocompromised patients developing NSTIs have a higher risk of dying and different characteristics than non-immunocompromised patients, including less regional inflammatory signs and more frequent abdomino-perineal topography and gram-negative bacilli documentation [6–8]. Previous studies on NSTIs in immunocompromised patients are scarce, consisted in case series or small cohort studies, and included patients with any kind of immunosuppression, thereby limiting the generalizability of the data in this highly heterogeneous population.

Neutropenic patients with NSTIs have been shown to exhibit a high mortality with frequent initial misdiagnosis associated with mild symptoms. The clinical and microbiological features of NSTIs in neutropenic patients are poorly known. Moreover, there are major gaps of knowledge in this specific patient population regarding the efficacy of key early therapeutic interventions. These include surgical debridement, which has sometimes been considered as an aggressive treatment with a questionable benefit-to-risk ratio in neutropenic patients [7, 9], the use of granulocyte colony-stimulating factor (G-CSF) or of granulocyte transfusion.

We thus performed a multicentre retrospective cohort study of neutropenic patients with NSTI hospitalized in the ICU, aiming at (1) describing their epidemiological, clinical and microbiological features, in comparison with a multicentre cohort of non-neutropenic NSTI patients, and (2) assessing the relationship between early therapeutic interventions and hospital mortality.

## Methods

### Study design and participants

We conducted a retrospective multicentre cohort study, including patients hospitalized in one of the 18 participating ICUs in France between 2011 and 2021. Participating centres are members of the GRRR-OH (Groupe de Recherche Respiratoire en Réanimation Onco-Hématologique) research network. This study was set in compliance with the Helsinki Declaration and was approved by the ethical committee of the French Intensive Care Society (CE SRLF n°21-79). Owing to the retrospective and observational nature of the study, patient’s consent was waived as per French law.

Patients were identified in each centre through a search in the medical database using the following keywords: “soft tissue infections”, “necrotizing soft tissue infections”, “necrotizing fasciitis”, “necrotizing skin infection”, “gas gangrene”, “gangrene” and “Fournier’s gangrene”. Patients were included in this study if they were hospitalized in ICU and had a proven or probable NSTI associated with a blood neutrophil counts < 1500/mm^3^ at the time of diagnosis. Proven NSTI was defined as surgically or histologically confirmed NSTI. Because surgery may not have been performed in all neutropenic patients with NSTIs, an expert committee (R.A. and N.d.P.) reviewed the medical files of patients without histological/surgical proof of necrosis and defined probable NSTI as follows: (a) patients with specific clinical signs of NSTI (i.e. skin necrosis or subcutaneous crepitation) and/or (b) patients with septic shock and/or bacteraemia without any other identified source of infection than skin and soft tissue.

Clinical characteristics were compared between neutropenic and non-neutropenic patients, including long-term steroid use, defined as steroid treatment for three months or more before NSTI onset, non-steroidal anti-inflammatory drug (NSAID) use, defined as any NSAID exposure during the week preceding NSTI onset, and immunosuppression, defined as exposure to any immunosuppressive drug or the presence of an underlying disease altering the immune system (i.e. haematological malignancies, asplenism, HIV infection, congenital or acquired immune deficits). We also identified the nosocomial status of infection, which was defined as a NSTI occurring after at least 48 h of hospitalization.

Microbiological documentation was based on blood cultures and/or on intraoperative deep tissue biopsy cultures or on the culture of fine-needle subcutaneous aspiration in an area of skin necrosis. Superficial skin swabs were not considered.

A previously published cohort of 165 non-neutropenic patients with histologically confirmed NSTIs admitted from 2015 to 2019 in the ICUs of 13 Great Paris area hospitals was used for comparison [10]. These patients were selected using the French national hospital database with the 10th International Classification of Disease diagnostic codes for “necrotizing skin and soft tissue infections”. Patients with cervico-facial NSTIs were not included in this cohort.

### Statistics

Descriptive results are presented as medians (interquartile range [IQR]) for continuous variables without normal distribution and as mean ± standard deviation for continuous variables with normal distribution and as numbers with percentages for categorical variables. Univariable comparisons were performed using Student’s *t* test or Mann–Whitney test for continuous variables, Chi2 or Fisher’s exact tests for categorical variables and Log-rank test for survival analysis, as appropriate. A *p*-value < 0.05 was considered as significant. Neutropenic and non-neutropenic patients were compared using aggregated data, due to the impossibility to merge individual data originating from these two populations in one data frame. We performed Cox proportional hazard models to assess the relationship between surgery and G-CSF administration and the risk of hospital death with G-CSF and surgery managed as time-dependent variables. Multivariable models were built, entering variables associated with a *p* value < 0.20 in univariable analysis and then applying a stepwise backward approach by only retaining variables statistically significant at *p* < 0.05 and those previously shown to be important confounding factors, including age and SAPS II, computing adjusted hazard ratios (aHR) and their 95% confidence interval (CI). Assumption for log-linearity of continuous variables and proportional hazard assumptions were checked for the final survival models. To further explore the influence of G-CSF on hospital death, we performed a propensity score overlap weighting analysis, which allowed for weighting patients from each treatment group with the probability of being assigned to the other treatment group. This model has demonstrated high stability and the ability to obtain precise adjustment in various situations, emphasizing the proportion of the population where the most treatment equipoise exists [11, 12]. The propensity score was built using logistic regression according to variables associated with G-CSF. Covariates for the G-CSF model included mechanical ventilation, shock, acute kidney injury (defined using KDIGO stage [13]), nosocomial NSTI, multifocal NSTI, chronic hypertension, surgery, abdominal localization and age. Quality of matching was assessed using propensity score distribution before and after overlap weighting. To further assess the robustness of our results, we performed as sensitivity analysis a complex bootstrap resampling [14]. First, the bootstrapping technique resampled the original set 10,000 times with replacement. Then, in each set, we assessed the unadjusted risk for mortality. Statistical analyses were performed using R version 3.6.2 (R Foundation for Statistical Computing), “survival”, “survey”, “ggplot2” and “PSweight” packages.

## Results

A total of 76 NSTI neutropenic patients were identified during the 10-year study period. For the majority of patients, neutropenia was associated with haematological malignancy (*n* = 58, 76.3%) or solid cancer (*n* = 8, 10.5%), while others suffered from various causes of neutropenia (drug adverse events, *n* = 6; hemophagocytic lymphohistiocytosis, *n* = 1; HIV infection, *n* = 1, others, *n* = 2) (Additional file 1: Table S1).

### Neutropenic patients showed a different picture of NSTI than non-neutropenic patients

As compared with a cohort of 165 non-neutropenic patients with NSTI, neutropenic patients were younger and had less cardiovascular and metabolic comorbidities (obesity, diabetes and peripheral arterial occlusive disease), while cancer and long-term corticosteroid treatment were more frequent (Table 1). NSAIDs use before NSTI diagnosis was more frequent in non-neutropenic patients.

**Table 1:** Demographics and characteristics of patients with NSTI, according to the presence of neutropenia

	Non-neutropenic patients (*N* = 165)	Neutropenic patients (*N* = 76)	*p*-value*
General characteristics			
Age, years	60.1 ± 13.0	54.2 ± 14.1	**0.002**
Sex			
Male	118 (71.5)	48 (63.2)	0.193
Female	47 (28.5)	28 (36.2)	
Obesity	58/165 (35.2)	12/65 (18.5)	**0.01**
Diabetes	92 (55.8)	9 (11.8)	**< 0.001**
PAOD	26 (15.8)	1 (1.3)	**0.001**
Liver cirrhosis	15/164 (9.1)	2/76 (2.6)	0.067
HIV infection	4 (2.4)	5 (6.6)	0.145
Cancer *	10 (6.1)	67 (87)	**< 0.001**
COPD	17/164 (10.4)	7/76 (9.2)	0.792
ESRD requiring dialysis	5 (3.0)	1 (1.3)	0.668
Alcohol consumption	49 (29.7)	9 (11.8)	**0.002**
Long-term steroid therapy	25 (15.2)	21 (27.6)	**0.022**
NSAIDs	35/163 (21.5)	1/76 (1.3)	**< 0.001**
SAPS II	47.5 ± 22.7	62.1 ± 21.9	**< 0.001**
Characteristics of NSTI			
Hospital-acquired	38 (23.0)	43 (56.6)	**< 0.001**
NSTI location			
Upper Limbs	24 (14.6)	11 (14.5)	0.988
Lower limbs	117 (70.9)	34 (44.7)	**< 0.001**
Abdomino-perineal	31 (18.8)	33 (43.4)	**< 0.001**
Cervical	–	7(9.2)	**–**
Thoracic	–	3 (3.9)	–
Monomicrobial documentation	60/149 (40.3)	50/76 (65.8)	**< 0.001**
Positive blood culture	49 (29.7)	47 (61.8)	**< 0.001**
Surgery	165 (100)	55 (72.4)	**< 0.001**
Amputation	26/161 (16.2)	4/76 (5.3)	**0.022**
IVIg	12/161 (7.5)	2/76 (2.6)	0.236
Hyperbaric oxygenotherapy	3/161 (1.9)	1/76 (1.3)	> 0.999
ICU outcomes			
Shock during ICU stay	125/162 (77.2)	65/76 (85.5)	0.166
Invasive mechanical ventilation during ICU stay	83/161 (51.6)	59/76 (77.6)	**< 0.001**
ICU length of stay	15.3 ± 20.8	14.1 ± 14.9	0.61
ICU mortality	35 (21.2)	39 (51.3)	**< 0.001**
Hospital length of stay	49.0 ± 45.1	30.0 ± 30.8	**< 0.001**
In-hospital mortality	47 (28.5)	44 (57.9)	**< 0.001**

Continuous variables are shown as mean ± standard deviation. Qualitative variables are shown as numbers (percentage). Long-term steroid use: ≥ 3 months treatment; * haematological malignancy or solid tumour; Bolded values are significant at the *p* < 0.05 level

At ICU admission, neutropenic patients presented with more severe disease, as illustrated by significantly higher SAPS II score (Table 1). During ICU stay, they required more frequent invasive mechanical ventilation support than others, but there was no significant difference between groups regarding need for vasopressor.

Regarding clinical and microbiological characteristics, neutropenic patients had more frequent nosocomial NSTIs [56.6% (*n* = 43/76) vs 23.0% (*n* = 38/165), *p* < 0.001] and associated bacteraemia [61.8% (*n* = 47/76) vs 29.7% (*n* = 49/165), *p* < 0.001] than their non-neutropenic counterparts. NSTIs were significantly more frequently localized in the abdomino-perineal region in neutropenic than in non-neutropenic patients (43.4% (*n* = 33/76) vs 18.8% (*n* = 31/165), *p* < 0.001), while lower limbs were less frequently involved [44.7% (*n* = 34/76) vs 70.9% (*n* = 117/165), *p* < 0.001] (Table 1).

We then compared 149 non-neutropenic patients for whom microbiological data were available to the 76 neutropenic patients. We observed that *Streptococcus pyogenes*, other *Streptococcus* species and *Staphylococcus aureus* were significantly more frequently isolated in non-neutropenic than in neutropenic patients. In contrast, gram-negative bacilli were more frequently documented in neutropenic patients, who also had more monomicrobial infections. Non-fermenting gram-negative bacilli, mainly *Pseudomonas aeruginosa* and *Stenotrophomonas maltophilia*, were more frequently isolated in neutropenic than in non-neutropenic patients (Table 2). The resistance profile of 16/24 *Pseudomonas aeruginosa* isolates was available: 12/16 (75%) were categorised as wild, 2/16 (12.5%) were carbapenem-resistant and 2/16 (12.5%) were pan-resistant for beta-lactams. For patients infected with Enterobacterales the resistance profiles were available for 22/36 isolates: 11/22 (50%) were categorised as wild, and 4/22 (18.2%) carried an extend-spectrum beta-lactamase (ESBL).

**Table 2:** Microbiological documentation in NSTIs according to neutropenic status

Isolated bacteria	Non-neutropenic patients	Neutropenic patients	*p*-value
	*N* = 149	*N* = 76	
Gram-negative bacteria			
Enterobacterales	52 (34.9)	34 (44.7)	0.15
* Escherichia coli*	23 (15.4)	19 (25)	0.08
* Enterobacter cloacae*	6 (4)	3 (3.9)	
* Klebsiella pneumoniae*	10 (6.7)	4 (5.3)	
* Proteus mirabilis*	11 (7.4)	1 (1.3)	
* Citrobacter spp*	5 (3.4)	2 (2.6)	
* Klebsiella oxytoca*	3 (2)	0 (0)	
* Morganella morganii*	3 (2)	0 (0)	
* Serratia marcescens*	4 (2.7)	1 (1.3)	
Unprecised		6 (7.9)	
Non-fermenting bacteria	26 (17.4)	29 (38.2)	**< 0.001**
* Pseudomonas aeruginosa*	24 (16.1)	24 (31.6)	**0.007**
* Acinetobacter spp.*	4 (2.7)	1 (1.3)	
* Pseudomonas stutzeri*	0 (0)	1 (1.3)	
* Stenotrophomonas maltophilia*	0 (0)	3 (3.9)	
Gram-positive bacteria			
* Streptococcus pyogenes*	38 (25.5)	0 (0)	**< 0.001**
Other streptococcus species	47 (31.5)	8 (10.5)	**< 0.001**
* Streptococcus agalactiae*	10 (6.7)	0 (0)	
* Streptococcus anginosus*	12 (8.1)	0 (0)	
* Streptococcus constellatus*	5 (3.4)	1 (1.3)	
* Streptococcus oralis*	4 (2.7)	0 (0)	
* Streptococcus dysgalactiae*	5 (3.4)	1 (1.3)	
* Enterococcus faecium*	8 (5.4)	3 (3.9)	
* Enterococcus faecalis*	14 (9.4)	2 (2.6)	
* Enterococcus durans*	0 (0)	1 (1.3)	
* Staphylococcus aureus*	38 (25.5)	3 (3.9)	**< 0.001**
* Staphylococcus coag neg*	7 (4.7)	6 (7.9)	
Anaerobic bacteria	15 (10.1)	6 (7.9)	0.59
Other	11 (7.4)	5 (6.6)	0.82
Undocumented	–	7 (9.2)	

NSTI management was also strikingly different between neutropenic and non-neutropenic patients. Surgery was performed for all non-neutropenic patients (per inclusion criteria), while it was performed in only 72.4% (*n* = 55/76) of neutropenic patients with more amputation performed in the former than in the latter [16.2% (*n* = 26/161) vs 5.3% (*n* = 4/76), *p* = 0.022]. Adjuvant therapy using IVIg and hyperbaric oxygenotherapy was not significantly different between groups.

In-hospital mortality was significantly higher in neutropenic than in non-neutropenic patients [57.9% (*n* = 44/76) *vs* 28.5% (*n* = 47/165); *p* < 0.001].

### Characteristics and management of patients with neutropenic NSTIs

We further assessed the characteristics of neutropenic patients according to their in-hospital vital status (Additional file 1: Table S2). NSTI diagnosis was made after a median time of 4 [1; 14] days after neutropenia onset, which was not different between survivors and deceased patients. At the time of diagnosis median neutrophil counts was 0.1 [0; 0.5] G/L. Antibiotic use, NSTI topography and microorganisms involved did not differ significantly between groups.

Overall, 43.4% of patients (*n* = 33/76) received G-CSF. The median time elapsed between NSTI diagnosis and G-CSF administration was 1 [0.0; 2.75] days in survivors and − 1.0 [− 7.0; 1.0] days in decedents (*p* = 0.140). Other adjuvant therapies included granulocytes transfusion, which was used in 15.8% of patients (*n* = 12/76), intravenous immunoglobulins in 2.6% (*n* = 2/76) and hyperbaric oxygenotherapy in 1.3% (*n* = 1/76) with no significant difference between survivors and decedents (Additional file 1: Table S2).

### Impact of G-CSF administration and surgery on hospital survival of neutropenic NSTI patients

**Fig. 1:**
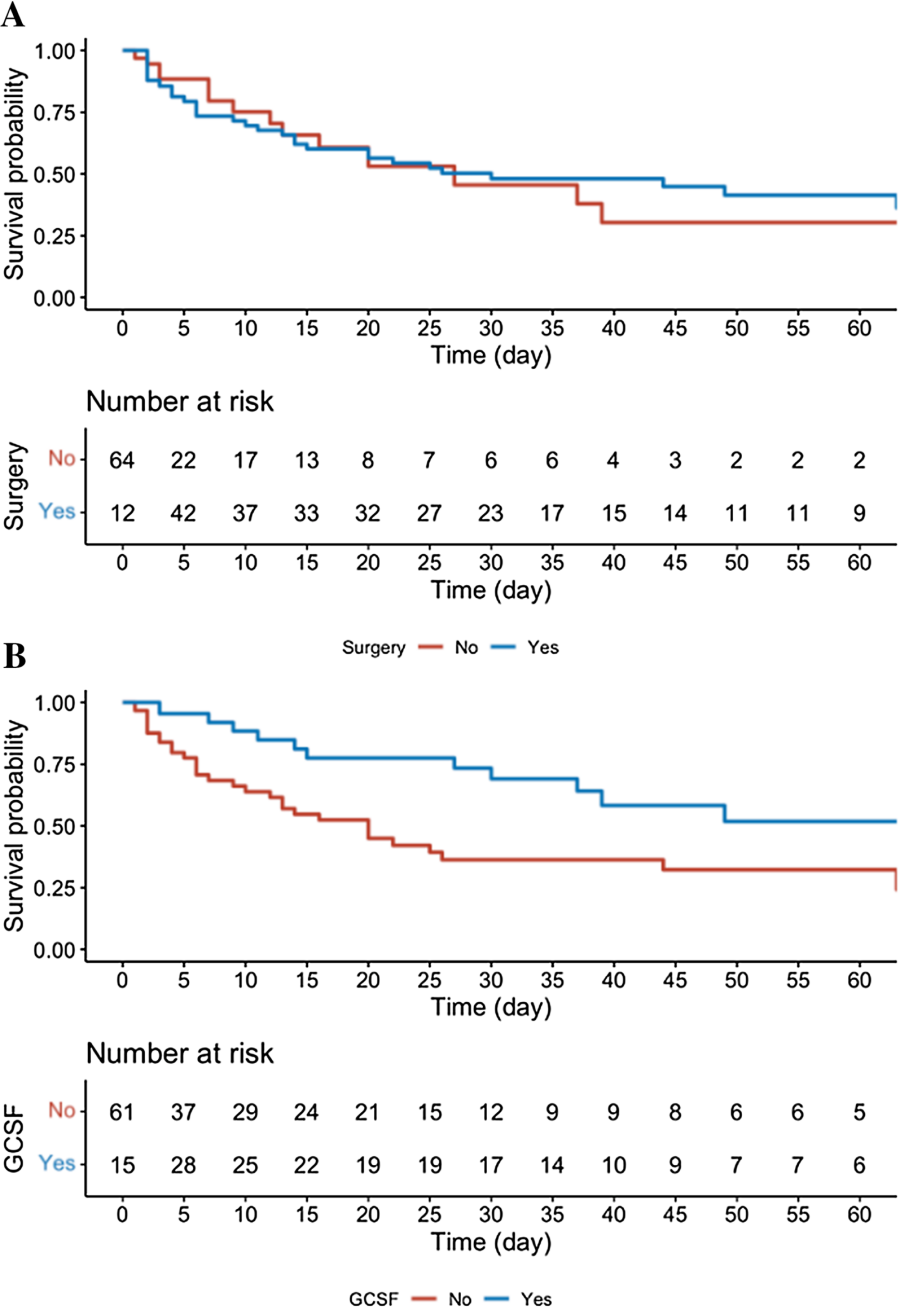
Univariable Kaplan–Meier curves of the probability of survival according to surgery (**A**) and granulocyte colony-stimulating factor (G-CSF) treatment (**B**). Blue lines indicate patients who received the treatments (surgery in panel **A**, G-CSF in **B**); red lines indicate patients who did not receive the treatments (no surgery in **A**, no G-CSF in **B**)

We further explored whether surgery and G-CSF administration had a prognostic impact in neutropenic NSTI patients. In univariable Cox analysis, surgery was not significantly associated with in-hospital mortality (HR = 0.81 95% CI [0.46–1.4]; *p* = 0.48). In contrast, in univariable and multivariable Cox analyses, adjusting for important outcome confounders in NSTIs [15–17], G-CSF administration was significantly associated with in-hospital survival (aHR = 0.46 95% CI [0.22–0.94], *p* = 0.033) (Table 3**, **Fig. 1). Importantly, G-CSF was administered with a median time interval of 0 [− 2;2] days from NSTI diagnosis. We used overlap propensity score weighting to further assess the association between G-CSF and in-hospital mortality. After overlap propensity score weighting, both groups were adequately balanced (Additional file 1: Figure S1), and G-CSF remained protective for in-hospital mortality (overlap propensity score-weighted odds ratio (OR) = 0.25 95% CI [0.09; 0.68], *p* = 0.006) (Additional file 1: Figure S2). We also used a bootstrapping technique, resampling the original set 10,000 times for mortality risk according to G-CSF use. The results were consistent with univariate survival analysis, suggesting our findings may not be ascribable to outliers (Additional file 1: Figure S3).

**Table 3:** Univariable and multivariable Cox analyses of factors associated with in-hospital mortality in neutropenic NSTI patients (n = 76)

	In-hospital mortality			
	Univariable		Multivariable	
	HR [95% CI]	*p*-value	aHR [95% CI]	*p*-value
Model 1				
Surgery	0.81 [0.46–1.4]	0.48	–	
G-CSF	0.43 [0.23–0.82]	**0.010**	0.46 [0.22–0.94]	**0.033**
SAPSII	1 [1–1]	**< 0.001**	1.03 [1.01–1.04]	**< 0.001**
Age, years	1 [1–1]	**0.03**	1.03 [1.01–1.06]	**0.017**
Abdomino-perineal location	0.63 [0.35–1.1]	0.13	0.52 [0.28–0.98]	**0.042**

HR [95%CI]: hazard ratio [95% confidence interval]; aHR [95%CI]: adjusted hazard ratio [95% confidence interval]; G-CSF: granulocyte colony-stimulating factor; ^a^Missing values in 4 patients have been imputed. **Bolded** values are significant at p < 0.05

Finally, as a sensitivity analysis, we tested the effect of G-CSF in the subgroup of neutropenic patients who underwent surgery (*n* = 55) and confirmed G-CSF was still significantly associated with survival (Additional file 1: Figure S4).

## Discussion

In this multicentre retrospective cohort study, we show that NSTIs in neutropenic patients have specific features: (1) More frequent abdomino-perineal location, nosocomial infections, bacteraemia; (2) Less *Streptococcus pyogenes* and more *Pseudomonas aeruginosa* documentation than non-neutropenic patients; (3) Higher ICU and in-hospital mortality than patients with non-neutropenic NSTIs and (4) G-CSF administration was associated with in-hospital survival.

To our knowledge, this is the largest cohort study describing the presentation and outcomes of critically ill neutropenic patients with necrotizing soft tissue infections. Previous studies were either small case series or included a wide spectrum of immunocompromised patients [6, 7]. We reported a higher mortality in neutropenic NSTI patients, consistent with previous studies in immunocompromised patients. Such outcome difference between neutropenic and non-neutropenic patients is probably in part accounted for by the poor prognosis of the underlying disease causing neutropenia, with a large majority of patients suffering from haematological malignancies. Indeed, the SAPS II score at ICU admission was higher in neutropenic patients than in others despite similar rates of vasopressor needs during ICU stay. Moreover, neutropenic patients had more frequent bacteraemia, indicating more frequent invasive infections. We could identify the cause of death in 39/44 neutropenic patients. Among these, 27 died directly from a septic shock related to NSTI (69.2%), while others died from various complications. Despite more nosocomial infections and non-fermenting gram-negative bacilli documentation in neutropenic than in non-neutropenic patients, the empirical antibiotic treatment was effective in most cases (89.9%).

Immunosuppression was identified as a risk factor for non-streptococcal NSTI in a previous study [4]. Our results showing a predominance of gram-negative bacteria documentation in neutropenic patients confirm that a broad-spectrum empiric antibiotherapy needs to include coverage for these pathogens, particularly *Pseudomonas aeruginosa,* which was the most frequently isolated non-fermenting gram-negative bacteria. Unfortunately, multi-drug resistant bacteria colonization status was unavailable for the majority of the patients due to the retrospective nature of the study. Yet, the risk of ESBL-producing Enterobacterales infection should be considered on a case by case basis in the empirical antibiotic regimen [18].

In line with previous reports [6], we observed that neutropenic patients were less likely to undergo a surgical debridement and required less amputation, which is probably partially explained by the lower rate of limb infection. We did not observe a significant association between surgery and hospital survival. In neutropenic patients, 21 (27.6%) patients did not undergo surgery, of whom 16 (76.2%) died, while among the 55 surgically treated patients, 28 (50.9%) were dead at hospital discharge. Patients who were not surgically treated were either too severe to undergo surgery, presenting a refractory shock (*n* = 8/21,38.1%) or the decision to forgo surgery had been taken for ethical reasons (*n* = 6/21, 28.6%). Only 7/21 (33%) patients improved without surgery, of whom only 5 were alive at hospital discharge. Moreover, patients treated with surgery presented a wide heterogeneity of clinical presentation, some of them having a small area of cellulitis and necrosis and probably a better prognosis after surgery than others with larger areas of necrosis. Finally, considering our definition of probable NSTI and the fact that we could not analyse the histological findings for all patients we could hardly discriminate whether some neutropenic patients underwent a mere incision/drainage or had an associated tissue debridement, particularly when infections were located in abdomino-perineal regions. The lack of association between surgery and a positive outcome could thus be accounted for by unmeasured confounders reflecting the heterogeneity of surgery and debridement extension. Nevertheless, early surgery should probably be considered for each patient regarding the risk–benefit balance of invasive interventions in these patients at high risk of complicated outcomes.

We identified G-CSF treatment as associated with survival. G-CSF is recommended in primary or secondary prophylaxis subsequently to chemotherapy at high risk of neutropenia leading to a reduction of infection, infection-related mortality and all-cause mortality [19, 20]. Its use is controversial in patients with already established neutropenia, particularly in those with fever or infection. A meta-analysis showed that G-CSF did not improve mortality in this context despite a shorter duration of neutropenia and duration of antibiotic use [21], although ICU patients were not included. Therefore, the American Society of Clinical Oncology recommends using G-CSF in neutropenic patients with fever if already initiated before fever onset or in patients with febrile neutropenia at high risk of complication [22]. Importantly, in our study, the time from NSTI diagnosis to G-CSF administration was not statistically different between survivors and decedents.

Our study has some strengths, including its multicentre design, with participating centres having developed specific multidisciplinary care bundle for NSTI [23]. The comparison to a multicentre cohort of non-neutropenic NSTI patients allowed us to assess the clinical and microbiological characteristics of neutropenic patients [10].

This study certainly has limitations, inherent to its retrospective and observational design. We also could not retrieve the accurate clinical description of initial skin lesions for all patients, and therefore could not compare the clinical lesions of neutropenic and non-neutropenic patients. Then, we acknowledge that the association of G-CSF with survival could suffer from a risk of immortal time bias, although the delay between ICU admission and this therapeutic intervention was very short and we considered G-CSF as a time-dependent variable, thus making this risk theoretical. In our study we compared neutropenic patients to a previously described cohort of non-neutropenic patients [10]. The data of the non-neutropenic patients were obtained from a retrospective search in the French national hospital database in 13 centres from the Assistance Publique—Hôpitaux de Paris (APHP) network, while neutropenic patients were from 6 AP-HP centres and 12 other French hospitals. Importantly, not all these centres, particularly those managing neutropenic patients, have implemented multidisciplinary care bundles for NSTI [23], which may in part account for the lower rate of surgery and the poorer outcome of neutropenic patients. Moreover in the non-neutropenic cohort, patients with cervical infection were not included, which we acknowledge might have resulted in a selection bias.

## Conclusions

In ICU, neutropenic patients suffering from NSTIs have a higher mortality than non-neutropenic patients. Specific features of NSTIs in neutropenic patients are more frequent abdomino-perineal location with more frequent gram-negative bacteria. G-CSF was associated with lower in-hospital mortality and should be considered as soon as possible. Surgery was not associated with improved survival in our study but should probably remain the cornerstone of management of NSTIs in neutropenic as in non-neutropenic patients.

## Supplementary Information

**Additional file 1: Table S1.** Distribution of neutropenia causes in neutropenic patients with NSTIs.** Table S2.** Characteristics of patients with neutropenic NSTI according to in-hospital vital status.** Figure S1.** Effect of overlap weighting on covariate balance across patients exposed or not to G-CSF.** Figure S2.** Kaplan-Meier curves of the probability of survival according to G-CSF treatment after overlap weighting. Blue lines indicate patients who received the treatment; Red lines indicate patients who did not receive the treatment.** Figure S3.** Distribution of HR for G-CSF administration on hospital mortality according to bootstrapping analysis.** Figure S4.** Kaplan-Meier curves of the probability of survival according to G-CSF in patients who underwent surgery (n = 55). Blue lines indicate patients who received the treatment; Red lines indicate patients who did not receive the treatment.

## Data Availability

All data generated or analysed during this study are included in this published article and its supplementary files.

## Abbreviations

NSTI: Necrotizing soft tissue infection
ICU: Intensive care unit
G-CSF: Granulocytes colony-stimulating factor
NSAID: Non-steroidal anti-inflammatory drug
SAPSII: Simplified acute physiology score
PAOD: Peripheral arteritis occlusive disease
IMV: Invasive mechanical ventilation
ESRD: End stage renal disease
IVIg: Polyvalent intravenous immunoglobulin
