# Supplementary material for: Necrotizing soft tissue infections in critically ill neutropenic patients: a French multicentre retrospective cohort study
Source: Ann Intensive Care. 2023 Apr 28;13:34. doi: 10.1186/s13613-023-01125-w (PMC10147851; doi:10.1186/s13613-023-01125-w)

**Necrotizing soft tissue infections in critically ill neutropenic patients: a French multicenter retrospective cohort study**

Romain Arrestier^1,2,3^, Anis Chaba^4^, Asma Mabrouki^5^, Clément Saccheri^6^, Emmanuel Canet^7^, Marc Pineton de Chambrun^8^, Anabelle Stoclin^9^, Muriel Picard^10^, Florent Wallet^11^, François Perier^12^, Matthieu Turpin^13^, Laurent Argaud^14^, Maxens Decavèle^15^, Nahema Issa^16^, Cyril Cadoz^17^, Kada Klouche^18^, Johana Cohen^19^, Djamel Mokart^20^, Julien Grouille^21^, Tomas Urbina^22^, Camille Hua^23,24^, Olivier Chosidow^23^, Armand Mekontso-Dessap^1,2,3^, Elie Azoulay^5^, Nicolas de Prost^1,2,3^

Corresponding author:

Dr Romain Arrestier

Service de Médecine Intensive et Réanimation, Hôpital Henri Mondor, Créteil, France

Phone number: +33145178506

Mail: [romain.arrestier@aphp.fr](mailto:romain.arrestier@aphp.fr)

**Online supplement**

**Table of content**

Supplemental Table S1………………………………………………………………………………...2

Supplemental Table S2………………………………………………………………………………...3

Supplemental Figure 1..……………………………………………………………………….……….7

Supplemental Figure 2…………..………………………………………………………….………….8

Supplemental Figure 3…………..………………………………………………………….………….9

Supplemental Figure 4…………………...…………………………………………………………….10

**Table S1. Distribution of neutropenia causes in neutropenic patients with NSTIs**

| **Causes** | **N (%)** |
| --- | --- |
| **Hematological malignancy** | **58 (76.3%)** |
| - Acute myeloid leukemia | 24 (31.6%) |
| - Diffuse large B cell lymphoma | 12 (15.8%) |
| - Acute lymphoblastic leukemia | 6 (7.9%) |
| - Other lymphoma | 5 (6.6%) |
| - Burkitt lymphoma | 3 (3.9%) |
| - Follicular lymphoma | 2 (2.6%) |
| - Multiple myeloma | 2 (2.6%) |
| - Myeloproliferative syndrome | 1 (1.3%) |
| - Primitive myelofibrosis | 2 (2.6%) |
| - Hodgkin lymphoma | 1 (1.3%) |
| **Solid cancer** | **8 (10.5%)** |
| - Lung | 4 (5.3%) |
| - Breast | 1 (1.3%) |
| - Oesophagus | 1 (1.3%) |
| - Mesothelioma | 1 (1.3%) |
| - Glioblastoma | 1 (1.3%) |
| **Other** | **10 (13.1%)** |
| - Drug adverse events - Cyclophosphamide treatment - Systemic lupus erythematosus - Granumolatosis with polyangiitis | 6 (7.9%)  3 (3.9%)  2 (2.6%)  1 (1.3%) |
| - Methotrexate treatment (Crohn’s disease) - NSAID - Calcineurin inhibitor | 1 (1.3%)  1 (1.3%)  1 (1.3%) |
| - HIV infection | 1 (1.3%) |
| - Hemophagocytic lymphohistiocytosis | 1 (1.3%) |
| - Others | 2 (2.6%) |

NSAID: non-steroidal anti-inflammatory drug

**Table S2. Characteristics of patients with neutropenic NSTI according to in-hospital vital status**

|  |  | **Death during hospitalisation** | |  |  |
| --- | --- | --- | --- | --- | --- |
|  | **All patients (N= 76)** | **No (N= 32)** | **Yes (N= 44)** |  | ***p* value** |
| **General characteristics** |  |  |  |  |  |
| Age, years | 58 [46.0; 64.0] | 51 [36.8;62.3] | 59.5 [52;65] |  | **0.027** |
| Male gender | 48 (63.2) | 24 (75) | 24 (54.5) |  | 0.113 |
| BMI, kg/m² | 25.3 [22.3; 28.3] | 25.3 [21.9;27.1] | 24.5 [22.6;29.4] |  | 0.627 |
| Obesity | 12/65 (18.5) | 4/30 (13.3) | 8/35 (22.9) |  | 0.324 |
| Hypertension | 21 (27.6) | 7 (21.9) | 14 (31.8) |  | 0.486 |
| Diabetes | 9 (11.8) | 5 (15.6) | 4 (9.1) |  | 0.609 |
| PAOD | 1 (1.3) | 0 (0) | 1 (2.3) |  | >0.99 |
| Chronic pulmonary disease | 7 (9.2) | 3 (9.4) | 4 (9.1) |  | >0.99 |
| Chronic kidney disease | 3 (3.9) | 1 (3.1) | 2 (4.5) |  | >0.99 |
| ESRD requiring RRT | 1 (1.3) | 0 (0) | 1 (2.3) |  | >0.99 |
| Liver cirrhosis | 2 (2.6) | 0 (0) | 2 (4.5) |  | 0.620 |
| HIV infection | 5 (6.6) | 2 (6.2) | 3 (6.8) |  | >0.99 |
| Long term steroid therapy | 21 (27.6) | 11 (34.4) | 10 (22.7) |  | 0.389 |
| Alcohol consumption | 9 (11.8) | 3 (9.4) | 6 (13.6) |  | 0.835 |
| **Neutropenia characteristics** | | | |  |  |
| Neutropenia onset to diagnosis, days | 4.0 [1.0; 14.0] | 6 [1.8;15.5] | 3 [1;13.8] |  | 0.277 |
| ICU admission - neutropenia resolution, days | 5.0 [2.0; 9] | 4.5 [2.3;10.3] | 5.5 [2.3;8.5] |  | 0.920 |
| Neutrophils count at diagnosis (G/L) | 0.1 [0;0.5] | 0.2 [0;0.5] | 0.02 [0;0.5] |  | 0.960 |
| **NSTI characteristics and management** | | | | | |
| Antibiotherapy before ICU for NSTI | 58 (76.3) | 25 (78.1) | 33 (75) |  | 0.966 |
| Efficacy of empirical antibiotherapy | 62/69 (89.9) | 27/30 (90) | 35/39 (89.7) |  | >0.99 |
| Type of antibiotic |  |  |  |  |  |
| β-lactam | 76 (100) | 32 (100) | 44 (100) |  | >0.99 |
| Clindamycin | 25 (32.9) | 9 (28.1) | 16 (36.4) |  | 0.470 |
| Aminoglycoside | 58 (76.3) | 23 (71.9) | 35 (79.5) |  | 0.615 |
| Glycopeptides | 24/69 (34.8) | 8/27 (29.6) | 16/42 (38.1) |  | 0.610 |
| Nosocomial NSTI | 43 (56.6) | 17 (53.1) | 26 (59.1) |  | 0.777 |
| NSTI localization |  |  |  |  |  |
| Upper limbs | 11 (14.5) | 4 (12.5) | 7 (15.9) |  | 0.931 |
| Lower limbs | 34 (44.7) | 12 (37.5) | 22 (50) |  | 0.396 |
| Abdomino-perineal | 33 (43.4) | 16 (50) | 17 (38.6) |  | 0.452 |
| Cervical | 7 (9.2) | 3 (9.4) | 4 (9.1) |  | >0.99 |
| Thoracic | 3 (3.9) | 2 (6.2) | 1 (2.3) |  | 0.777 |
| Multifocal | 9 (11.8) | 3 (9.4) | 6 (13.6) |  | 0.835 |
| Bacteraemia | 47 (61.8) | 18 (56.2) | 29 (65.9) |  | 0.475 |
| Surgery | 55 (72.4) | 27 (84.4) | 28 (63.6) |  | 0.082 |
| Reason for non-surgical treatment |  |  |  |  | **0.010** |
| Clinical improvement with medical therapy only | 7 (9.2) | 5 (15.6) | 2 (4.5) |  |  |
| Refractory shock | 8 (10.5) | 0 (0) | 8 (18.2) |  |  |
| Do not resuscitate order | 6 (7.9) | 0 (0) | 6 (13.6) |  |  |
| Amputation | 4 (5.3) | 3 (9.4) | 1 (2.3) |  | 0.396 |
| Time from NSTI diagnosis to surgery, days | 1.0 [0.2; 2.0] | 2 [1;3] | 1 [0;1] |  | 0.073 |
| G-CSF administration | 33 (43.4) | 19 (59.4) | 14 (31.8) |  | **0.031** |
| Time from NSTI diagnosis to G-CSF, days | 0 [-2.0;2.0] | 1 [0;3] | -1 [-7;-1] |  | 0.140 |
| Granulocytes transfusion | 12 (15.8) | 7 (21.9) | 5 (11.4) |  | 0.356 |
| IVIg | 2 (2.6) | 0 (0) | 2 (4.5) |  | 0.620 |
| Hyperbaric oxygenotherapy | 1 (1.3) | 1 (3.1) | 0 (0) |  | 0.872 |
| **ICU characteristics and outcomes** | | | | | |
| SAPS 2 | 58.0 [45.0; 75.0] | 49.5 [40;60.3] | 69.5 [51.5;85] |  | **<0.001** |
| SOFA score | 11 [7.0; 13.0] | 9 [5;11.3] | 11.5 [9;15] |  | **0.002** |
| Shock at ICU admission | 59 (77.6) | 23 (71.9) | 36 (81.8) |  | 0.454 |
| Shock during ICU stay | 65 (85.5) | 24 (75) | 41 (93.2) |  | 0.058 |
| Days of vasopressor | 3.0 [2.0; 6.0] | 4 [2;7.3] | 3 [2;5] |  | 0.177 |
| ECMO at ICU admission | 3 (3.9) | 0 (0) | 3 (6.8) |  | 0.363 |
| ECMO during ICU stay | 4 (5.3) | 1 (3.1) | 3 (6.8) |  | 0.848 |
| Time from ECMO implantation to NSTI diagnosis | -6.0 [-14.5; 0.0] | 0 [0;0] | -12 [-17;-6] |  | 0.346 |
| IMV at ICU admission | 49 (64.5) | 15 (46.9) | 34 (77.3) |  | **0.013** |
| IMV during ICU stay | 59 (77.6) | 21 (65.6) | 38 (86.4) |  | 0.062 |
| Days of IMV | 5.5 [2.0; 14.0] | 6 [2;14] | 4 [2;13.5] |  | 0.468 |
| AKI at ICU admission | 55 (72.4) | 22 (68.8) | 33 (75) |  | 0.732 |
| AKI during ICU stay | 58 (76.3) | 22 (68.8) | 36 (81.8) |  | 0.294 |
| RRT during ICU stay | 22 (28.9) | 8 (25) | 14 (31.8) |  | 0.696 |
| Lactate at ICU admission, mmol/L | 3.3[1.9; 6.6] | 3.1 [1.5;5.2] | 3.4 [2.1;8] |  | 0.212 |
| CPK at ICU admission, UI/L | 148 [62; 298] | 147 [57;210] | 151 [72;315] |  | 0.481 |
| Fibrinogen at ICU admission, g/L | 5.0 [3.0; 6.9] | 5.2 [3.8;6.7] | 5 [2.9;7.0] |  | 0.469 |
| Platelets at ICU admission, G/L | 29 [13; 49] | 22 [10;43] | 34 [14;56] |  | 0.119 |
| Hospital length of stay, days | 21 [7; 43.5] | 34.5 [20;58.5] | 11.5 [4;22.8] |  | **<0.001** |
| ICU length of stay, days | 8 [3; 20] | 16 [6;30.3] | 5 [2;14] |  | **0.001** |

Continuous variables are shown as median (interquartile range 25-75); Qualitative variables are shown as numbers (percentage); AKI, acute kidney injury; ECMO, extracorporeal membrane oxygenation; IMV, invasive mechanical ventilation; IVIg: Intravenous immunoglobulins; ICU: intensive care unit; RRT, renal replacement therapy. **Bolded** values are significant at the p<0.05 level.

**Figure S1. Effect of overlap weighting on covariate balance across patients exposed or not to G-CSF.**

**
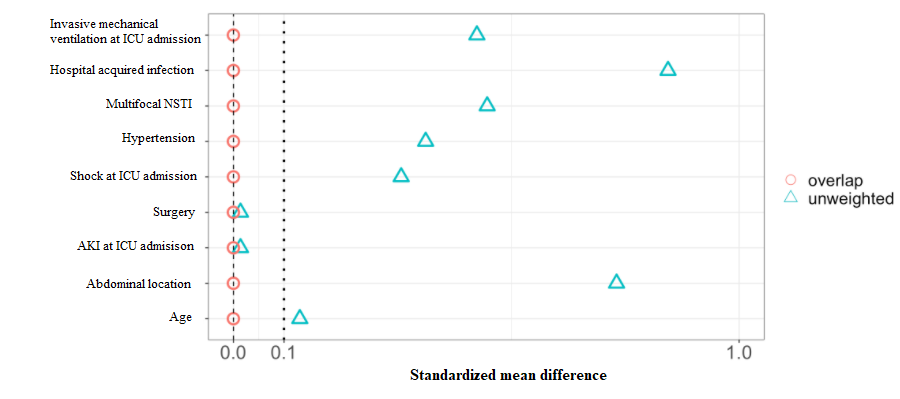
**

**Figure S2. Kaplan-Meier curves of the probability of survival according to G-CSF treatment after overlap weighting.** Blue lines indicate patients who received the treatment; Red lines indicate patients who did not receive the treatment

**
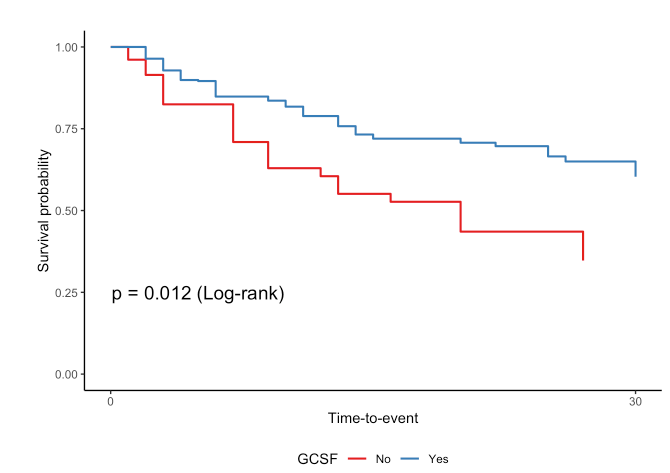
**

**Figure S3. Distribution of HR for G-CSF administration on hospital mortality according to bootstrapping analysis.**

**
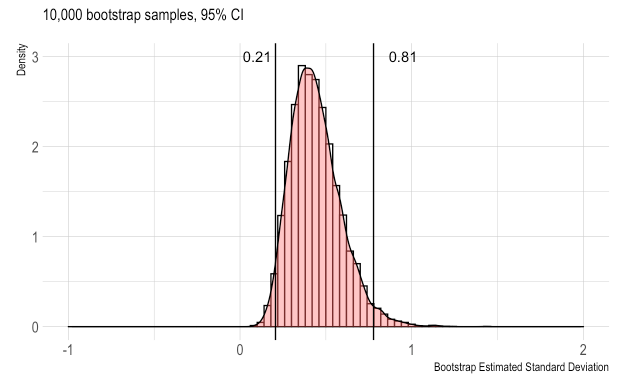
**

**Fig S4. Kaplan-Meier curves of the probability of survival according to G-CSF in patients who underwent surgery (n=55).** Blue lines indicate patients who received the treatment; Red lines indicate patients who did not receive the treatment


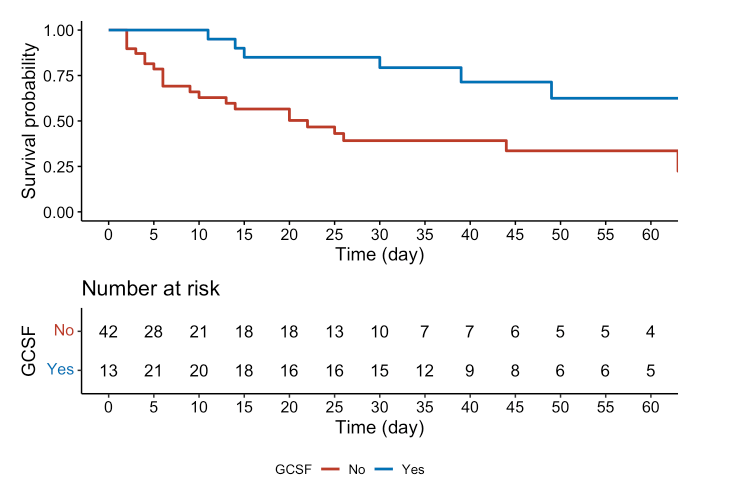

Supplement: Supplementary file 1 — Additional file 1: Table S1. Distribution of neutropenia causes in neutropenic patients with NSTIs. Table S2. Characteristics of patients with neutropenic NSTI according to in-hospital vital status. Figure S1. Effect of overlap weighting on covariate balance across patients exposed or not to G-CSF. Figure S2. Kaplan-Meier curves of the probability of survival according to G-CSF treatment after overlap weighting. Blue lines indicate patients who received the treatment; Red lines indicate patients who did not receive the treatment. Figure S3. Distribution of HR for G-CSF administration on hospital mortality according to bootstrapping analysis. Figure S4. Kaplan-Meier curves of the probability of survival according to G-CSF in patients who underwent surgery (n = 55). Blue lines indicate patients who received the treatment; Red lines indicate patients who did not receive the treatment. [file 13613_2023_1125_MOESM1_ESM.docx]
